# CD155: A Multi-Functional Molecule in Tumor Progression

**DOI:** 10.3390/ijms21030922

**Published:** 2020-01-30

**Authors:** Rosa Molfetta, Beatrice Zitti, Mario Lecce, Nadia Domenica Milito, Helena Stabile, Cinzia Fionda, Marco Cippitelli, Angela Gismondi, Angela Santoni, Rossella Paolini

**Affiliations:** Laboratory affiliated to Istituto Pasteur Italia–Fondazione Cenci Bolognetti, Department of Molecular Medicine, “Sapienza” University of Rome, “Viale Regina Elena 291, 00161 Rome, Italy; beatrice.zitti@ki.se (B.Z.); mario.lecce@uniroma1.it (M.L.); nadia.milito@uniroma1.it (N.D.M.); helena.stabile@uniroma1.it (H.S.); cinzia.fionda@uniroma1.it (C.F.); marco.cippitelli@uniroma1.it (M.C.); angela.gismondi@uniroma1.it (A.G.); angela.santoni@uniroma1.it (A.S.)

**Keywords:** tumor immune surveillance, Natural Killer (NK) cells, NK cell receptors and ligands

## Abstract

CD155 is an adhesion molecule belonging to the Nectin/Nectin-like family often overexpressed on tumor cells and involved in many different processes such as cell adhesion, migration and proliferation. In contrast to these pro-tumorigenic functions, CD155 is also a ligand for the activating receptor DNAM-1 expressed on cytotoxic lymphocytes including Natural Killer (NK) cells and involved in anti-tumor immune response. However, during tumor progression inhibitory receptors for CD155 are up-regulated on the surface of effector cells, contributing to an impairment of their cytotoxic capacity. In this review we will focus on the roles of CD155 as a ligand for the activating receptor DNAM-1 regulating immune surveillance against cancer and as pro-oncogenic molecule favoring tumor proliferation, invasion and immune evasion. A deeper understanding of the multiple roles played by CD155 in cancer development contributes to improving anti-tumor strategies aimed to potentiate immune response against cancer.

## 1. Introduction

CD155 is an immunoglobulin superfamily adhesion molecule involved in many different physiological processes ranging from cell adhesion and migration, proliferation and modulation of immune responses [1,2,3]. Based on its ability to mediate the binding of human poliovirus, CD155 was initially identified as PolioVirus Receptor (PVR) [4]. CD155 is also known as Necl5 since it is a member of the Nectins and Nectin-like (Necls) family of molecules that comprises four Nectins (Nectin1–4) and five Necls (Necl1–5) [1,5,6]. They are expressed in many different cell types and can function both as ligands and receptors, hence being able to bidirectionally signal between juxtaposed cells. Nectins and Necls mediate both homotypic and heterotypic adhesion between one cell and its neighbors or the extracellular matrix (ECM) components. They are connected to signaling pathways that control actin and microtubule dynamics and ultimately affect cell motility [5,6].

In particular, Nectins are involved in the organization of E-cadherin-based adherens junctions in epithelial cells through homophilic and/or heterophilic Ca^2+^-independent interactions and are linked with the cytoskeleton through a cytoplasmic domain that contains a motif able to bind the actin-binding protein afadin [7].

Differently from Nectins, Necl proteins, including CD155, lack this cytoplasmic motif. CD155 intracellular domain, instead, binds to Tctex-1, a light chain subunit of the dynein motor complex [8]. This interaction allows the retrograde axonal transport of CD155 containing endocytic vesicles [8]. Furthermore, CD155 is not involved in homophilic interactions but trans-interacts with Nectin3 on neighboring cells [9,10]. It also mediates cell-to-ECM adhesion by binding to the ECM protein vitronectin [11].

*CD155* gene transcription gives rise to the production of a mRNA that can be alternatively spliced into different isoforms and ultimately translated in four possible proteins: two transmembrane forms and two soluble forms [12,13]. All of them bear the same extracellular domains but the soluble secreted CD155 β and γ forms lack the transmembrane domain, whereas the two transmembrane isoforms of CD155, namely α and δ, differ in their intracellular tail. In particular, only the cytoplasmic domain of CD155α interacts with the μ1B subunit of the clathrin adaptor complex, directing the sorting of CD155α to basolateral membranes in epithelial cells [14]. Moreover, the CD155α isoform contains an Immunoreceptor Tyrosine-based Inhibition Motif (ITIM) responsible for signal transduction [3]. Upon antibody-mediated CD155 engagement, the ITIM motif is phosphorylated by the c-Src tyrosine kinase allowing the recruitment of the Src homology region 2 domain-containing phosphatase (SHP-2) that initiates intracellular signals [3,15]. 

Although constitutively expressed at low level in diverse healthy tissues including the kidney, lung, liver, and testes, CD155 isoforms are up-regulated in several types of human malignancies and their overexpression correlates with unfavorable prognosis [16,17,18,19,20,21]. Indeed, CD155 may favor proliferative signals and tumor growth along with cancer cell invasion and metastasis.

On the other hand, CD155 provides a direct link between cellular responses to stress and immune surveillance because it is a ligand for DNAX-associated molecule-1 (DNAM-1), an activating receptor expressed on Natural Killer (NK) cells and cytotoxic T cells [1,22]. Indeed, CD155 up-regulation renders tumor cells more sensitive to elimination by immune cells. Noteworthily, cytotoxic lymphocytes also express inhibitory receptors able to bind to CD155 [23], adding an additional level of complexity to the clinical significance of CD155 expression in cancer. 

**Figure 1:**
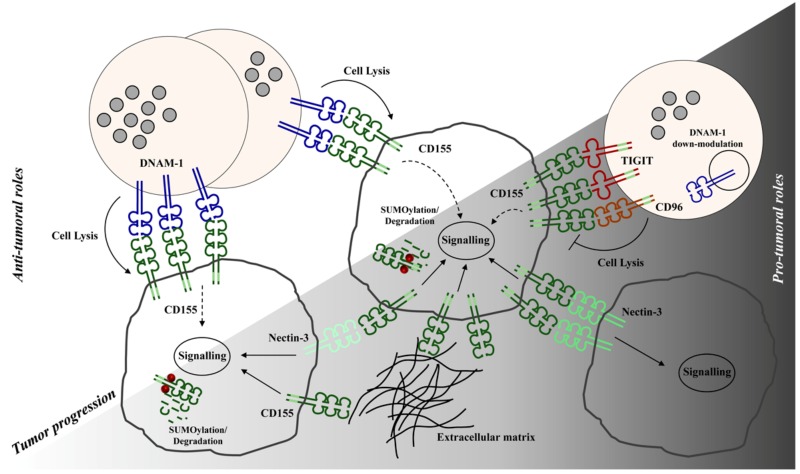
Model depicting CD155 multiple roles in tumor progression. Anti-tumoral and pro-tumoral CD155 roles are indicated with white and gray background, respectively. Temporal evolution of tumor is highlighted in shades of gray. CD155 represents an advantage for tumor cells (thick gray line) because its engagement initiates intracellular signals that favor proliferation, invasiveness and metastasis. However, in early phases of tumor transformation CD155 also plays an anti-tumorigenic role facilitating target recognition and killing by Natural Killer (NK) cells (light pink). For this reason, tumor cells employ different strategies (e.g., SUMOylation/Degradation) to reduce CD155 surface expression and to counteract DNAM-1-mediated recognition. In late phases, DNAM-1 down-modulation from NK cell surface and a concomitant up-regulation of inhibitory CD155 receptors (TIGIT and CD96) contribute to dampen anti-tumor immune responses.

In this scenario, it is likely that the role of CD155 will change during tumor progression: In the early phases of transformation CD155 surface expression on tumor cells mainly promotes anti-tumor immune function while in the late phases it supports tumor growth and immune escape (Figure 1). 

In this review, we will summarize data that have contributed to shedding light on the multifaceted roles of CD155 as pro-oncogenic adhesion molecule favoring tumor progression but also as a ligand for immune receptors regulating tumor immune surveillance. 

## 2. CD155-Mediated Signals Promote Tumor Progression

Several studies reported that cancer development is accompanied by up-regulation of CD155 expression that mainly occurs at transcriptional level in response to different stimuli [24,25]. 

Among signals implicated in malignant transformation, stimulation of Fibroblast Growth Factor receptor or oncogenic *ras* mutation activates a transcriptional program involving the Ras-Raf-MEK-ERK signaling pathway, ultimately leading to the induction of CD155 transcription [24].

Similarly, Sonic Hedgehog pathway, that is aberrantly active in many different tumors, has been shown to induce CD155 expression through the action of the transcription factor Gli [25]. 

As revealed by several lines of in vitro evidence, CD155 up-regulation may represent an advantage for tumor growth [5,6,26,27,28]. Accordingly, in Ras-mutated cells CD155 overexpression shortens the G0/G1 phase and contributes to tumor cell proliferation [26]. Although the signaling molecules involved have not been identified, yet, the cytoplasmic ITIM is required for CD155-induced proliferative signals, indicating that this function is exclusive for the CD155α isoform. 

CD155-mediated signaling may also cooperate with signals derived from growth factors to ultimately control tumor growth. For example, in NIH3T3 cells, CD155 enhances platelet-derived growth factor (PDGF)-induced cell proliferation potentiating the Ras-Raf-MEK-ERK signaling pathway [27].

In accordance with these findings, CD155 has been involved in proliferation and survival abilities of human colorectal cancer cells [28]. Indeed, CD155 knockdown suppresses proliferation of colon cancer cells and promotes apoptosis by affecting the ratio between Bax and Bcl-2 expression [28].

Regarding cell adhesion/migration, the ITIM domain of CD155 is responsible for the recruitment of SHP-2 which is activated and in turn dephosphorylates the focal adhesion kinase (FAK), ultimately resulting in increased cell motility [15,29].

In migrating cells, CD155 is recruited to the leading edge, colocalizes with actin and αvβ3 integrin, and activates CDC42 and Rac promoting actin reorganization, filopodia and lamellipodia formation [30]. In line with these evidences, CD155 expression on glioma cells enhances cell dispersal both in vitro and in primary brain tissue by the disassembly of focal adhesions [31].

All together these findings implicate CD155 as a negative regulator of adhesion signaling and a promoter of an invasive phenotype. 

**Figure 2:**
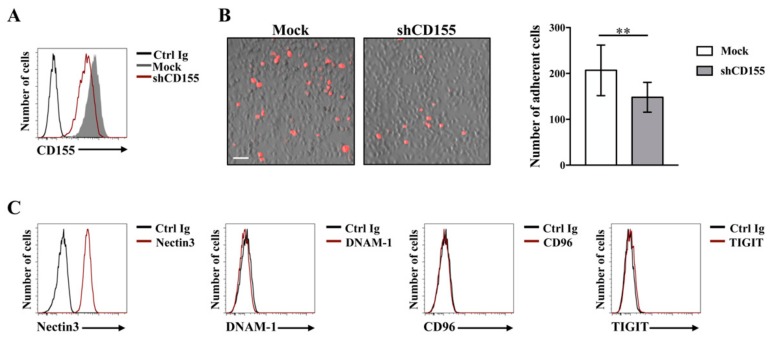
CD155 promotes Multiple Myeloma (MM) cell binding to bone marrow stromal cells (BMSCs). (**A**) CD155 knock-down in the Multiple Myeloma cell line ARK was achieved by means of lentiviral-mediated shRNA targeting (pLKO vector, MISSION™ Sigma-Aldrich). CD155 surface expression was analyzed by FACS in cells infected with shCD155 (code SHCLND-NM_006505, sequence TRCN0000062911) (empty red histogram) or non-targeting shRNA (Mock, filled gray histogram) by mean of a PE-conjugated anti-CD155 monoclonal Ab (clone SKII.4, Biolegend). Isotype control staining is also shown (Ctrl Ig, empty black histogram). Data from one representative experiment out of three independent experiments is shown. (**B**, left panel) CD155-silenced or mock-infected ARK cells were labeled with the red fluorescent dye PKH26 and left to adhere to a monolayer of HS5 bone marrow-derived stromal cells for 2 h at 37 °C. Cells were washed three times with PBS and fixed. Adherent cells were visualized with IX73 microscope equipped with a 10 X objective (Olympus). An overlay image mixing red fluorescence and brightfield is shown, scale bar: 50 μm. (**B**, right panel) Adherent cells were quantified with FIJI software. Means ± SD of 15 randomly acquired fields from two independent experiments are shown. ** *p* < 0.01, Unpaired t-test. (**C**) HS5 stromal cell line were stained for Nectin3 (clone N3.12.4, Millipore), DNAM-1 (clone DX11, Serotec), CD96 (clone NK92.39, Biolegend) and TIGIT (clone A15153G, Biolegend) (empty red histograms) or with isotype matched Ctrl Ig (black histograms) followed by an APC-conjugated goat anti-mouse Ab (Jackson Laboratories) and analyzed by flow cytometry. Data from one representative experiment out of three independent experiments is shown.

Interestingly, even though CD155 is involved in cell movement, its binding to Nectin3 on adjacent cells may facilitate cell-cell interactions [32]. Accordingly, we demonstrated that on Multiple Myeloma (MM), a hematopoietic tumor in which malignant plasma cells proliferate in the bone marrow niche, CD155 promotes MM cell adhesion to bone marrow stromal cells (BMSCs) (Figure 2). In particular, we found that shRNA-mediated CD155 knock-down dramatically decreases the number of MM/BMSC adherent cells (Figure 2A,B). Moreover, we also provided evidences that the only ligand of CD155 expressed on BMSCs is Nectin3 (Figure 2C), strongly suggesting its involvement in MM cell adhesion to stromal cells. Whether CD155/Nectin3 interaction also contributes to MM cell survival is under investigation. 

Previous data obtained on fibroblastic cells demonstrate that trans-interaction between CD155 and Nectin3 is rapidly followed by CD155 internalization resulting in contact inhibition of cell movement, thus promoting stable adhesion [32]. 

Although these in vitro evidences support a proto-oncogenic role for CD155 in tumor progression, the contribution of CD155 during tumor development in vivo is scarcely understood. 

CD155 deficient mice show reduced tumor development in a murine model of colitis-induced colorectal cancer [33]. Moreover, silencing of CD155 reduces proliferation of melanoma cells compared to control cells upon in vivo injection [34]. These findings, together with the high CD155 expression in advanced clinical stage of human malignancies including melanoma, glioblastoma, pancreatic, colon and lung cancers [16,17,18,19,20,21], support a role for CD155 as pro-tumorigenic molecule. 

However, CD155 overexpression may be exploited as a means to selectively target and eliminate malignant cells. Indeed, brain tumors overexpressing CD155 may become a target for oncolytic immunotherapy [35]. To this regard, an attenuated form of poliovirus that retains high cytolytic activity only in mitotically active cells is currently being tested for its ability to target and destroy CD155-positive glioblastoma cells [36]. 

## 3. CD155 is a Ligand for Immunoreceptors Implicated in Tumor Surveillance

CD155 also exerts an anti-tumorigenic role participating in immune response to tumors. Indeed, it is considered a stress-induced molecule able to activate a danger signal, alerting the immune system against tumor transformation. In particular, CD155 once up-regulated on different types of tumor cells is recognized by a group of receptors expressed on T and NK cells: The activating receptor DNAM-1 (CD226) and the inhibitory receptors TIGIT and TACTILE (CD96) [22,23,37]. 

It has been proposed that in the tumor microenvironment the balance between CD155/DNAM-1 and CD155/TIGIT/CD96 contrasting signals contributes to regulate NK cell effector functions [37].

### 3.1. Interaction of CD155 with DNAM-1 Activating Receptor 

DNAM-1 (also known as CD226) is an activating receptor that belongs to the Ig superfamily and is expressed on NK cells but also on T cells, monocytes and B cells. In this context, in addition to CD155 it also recognizes Nectin-2/CD112 [22]. DNAM-1 interaction with its ligands promotes serine phosphorylation of receptor cytoplasmic tail and the association with the integrin LFA-1 responsible for the activation of the Src family kinase Fyn [38], thus initiating signal transduction. Engagement of DNAM-1 co-stimulates CD8^+^ T cell and promotes NK cell cytotoxicity and cytokine production. In particular, on freshly isolated human NK cells, DNAM-1 requires the co-aggregation with at least another activating receptor to efficiently trigger the NK cell functional program [39].

DNAM-1/CD155 axis has raised interest in the context of anti-tumor immune response since DNAM-1-deficient mice are more prone to develop carcinogen-induced tumors compared to their wild type counterparts and exhibit accelerated CD155-positive transplanted tumor growth [40,41]. 

Additional in vivo evidences demonstrate a clear role for CD155 recognition by DNAM-1 in tumor immune surveillance in several murine models [42,43,44,45,46,47]. In mice injected with the RMA lymphoma cell line, the over-expression of CD155 results in DNAM-1-mediated tumor rejection by NK cells [42]. In addition, in a murine model of spontaneous MM development, DNAM-1 expression on both NK and T cells plays a prominent role in the control of tumor progression [47]. Instead, mice lacking DNAM-1 are more susceptible to lung metastases than wild-type mice [44,46], demonstrating a critical role for DNAM-1 in the control of tumor metastasis. Moreover, in a genetic model of spontaneous Burkitt lymphoma development, CD155 expression at early malignant stages mediates DNAM-1-dependent tumor cell elimination by NK and CD8^+^ T cells [45].

In humans, high CD155 levels on the surface of both solid and hematological tumors render them more susceptible to NK cell-mediated elimination in a DNAM-1-dependent manner [43,48,49,50,51,52]. Both CD155 and Nectin2 expression were found on neuroblastoma cells isolated from patients, and their levels correlate with tumor cell sensitivity to NK cell-mediated cytotoxicity [48]. However, only an anti-CD155 blocking antibody is able to interfere with NK cell killing, demonstrating that CD155 is the major DNAM-1 ligand. CD155 is also expressed by other solid tumors such as metastatic melanoma [43] and ovarian carcinoma [50] where it mediates NK cell recognition and tumor elimination.

In hematological malignancies, a dominant role of DNAM-1 receptor has been reported. Indeed, the NK cell activating ligands preferentially expressed in myeloid and lymphoid leukemias are the DNAM-1 ligands. Accordingly, NK cell-mediated leukemia cell elimination is largely impaired by the addition of an anti-DNAM-1 blocking antibody [49].

CD155 is also expressed on malignant plasma cells derived by the majority of MM patients and its recognition by DNAM-1 receptor contributes to NK cell-mediated malignant plasma cell elimination [51]. In this regard, strategies aimed to improve NK cell ability to kill MM cells, are based on the use of chemotherapeutic drugs that activate the DNA Damage Response (DDR) pathway and increase CD155 surface expression potentiating its transcription/expression [52,53,54,55,56]. However, whether CD155 α and δ transmembrane isoforms are equivalently able to bind DNAM-1 and activate cytotoxic program remains uninvestigated. 

The importance of CD155/DNAM-1 interaction is also supported by different tumor strategies aimed to counteract DNAM-1-mediated cancerous cell elimination. As mentioned above, tumor cells express different isoforms of CD155 including two soluble forms (sCD155), namely β and γ, both lacking the transmembrane region encoded by exon 6. CD155γ completely lacks exon 6, while CD155β contains a small exon fragment and for this reason is longer than the sCD155γ [13]. Since the extracellular domain of both soluble isoforms are identical to the extracellular domain of transmembrane CD155 forms, they are supposed to compete for DNAM-1 binding decreasing the efficacy of CD155/DNAM-1 activating signals and facilitating tumors to escape immune detection. In support to this hypothesis, sCD155 isoforms were found in blood serum, cerebrospinal fluid, and urine of patients with epithelial cancers at higher concentrations compared to healthy donors and correlate positively with disease stage [21]. Therefore, the presence of sCD155 isoforms can be considered a potential biomarker of tumor progression. In accordance with these findings, the amount of sCD155 produced by implanted cancerous cells in mice strongly correlates with the size of the resulting tumor [21]. However, whether the two CD155 soluble forms differ in their function is currently unknown.

**Figure 3:**
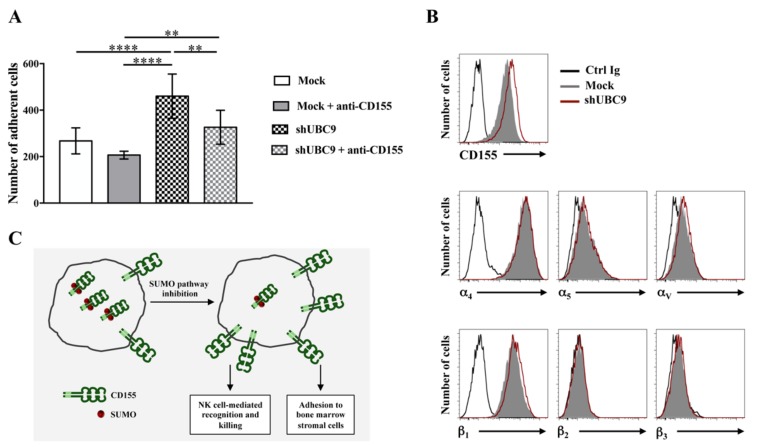
Inhibition of small ubiquitin-like modifier (SUMO) pathway increases MM adhesion to BMSCs. Knock down of the UBC9 SUMO conjugating enzyme in ARK cell line was obtained through shRNA-expressing lentiviral vectors, as previously described [63]. (**A**) UBC9 silenced or infected with the control vector pLKO non-targeting shRNA (Mock) ARK cells were labeled with the red fluorescent dye PKH26 and incubated for 20 min at 4 °C with anti-CD155 monoclonal Ab (clone D171, Thermo Scientific) or with isotype matched control Ab. After washing, cells were left to adhere to a monolayer of HS5 bone marrow-derived stromal cells for 2 h at 37 °C. Cells were washed three times with PBS and fixed. Adherent cells were visualized with IX73 microscope equipped with a 10X objective and quantified with FIJI software. Means ± SD of 10 randomly acquired fields from each experiment of three independent experiments are shown. **** *p* < 0.0001, ** *p* < 0.01, Two-way ANOVA. (**B**) UBC9 silenced (empty red histogram) and mock-infected (filled gray histogram) cells were analyzed by FACS for surface expression of CD155 and α/β integrin subunits using the following Abs: anti-α4 (clone P4G9, Telios Pharmaceuticals), anti-α5 (clone SAM-I, Immunotech), anti-αv (clone AMF7, Immunotech), anti-β1 (cloneTS2.16, generous gift from Dr. F. Sanchez-Madrid), anti-β2 (clone TS1.18, generous gift from Dr. F. Sanchez-Madrid) and anti-β3 (clone BB10, Chemicon) Abs. Isotype control staining is also shown (Ctrl Ig, empty black histogram). Data from one representative experiment out of two independent experiments is shown. (**C**) Working model illustrating how SUMO modification regulates CD155 expression and functions in MM cells.

CD155 surface expression on tumor cells may also be down-regulated by post-translational modifications [57], as previously shown for several immune receptors [58,59,60]. In hepatocellular carcinoma cells, the activation of the unfolded protein response promotes CD155 constitutive degradation and results in a defective NK cell activation against tumor cells [61]. Although not determined, it is likely that CD155 degradation depends on protein ubiquitination, as formally demonstrated for Nectin2 [62]. CD155 can also be covalently linked to the small ubiquitin-like modifier (SUMO) in different tumor cell lines, and this modification promotes CD155 intracellular retention [63]. Accordingly, inhibition of CD155 SUMOylation in tumor cells increases CD155 surface expression and improves NK cell surveillance [63]. Moreover, we provide evidence that silencing the SUMO conjugating enzyme UBC9 increases MM adhesion to BMSCs in a CD155-dependent manner (Figure 3). Indeed, the addition during the adhesion assay of the anti-CD155 monoclonal antibody D171, which reduces the binding to Nectin3 [10], partially inhibits tumor adhesion to stromal cells (Figure 3A). Several other adhesion molecules are implicated in MM adhesion to BMSCs including the α and β chain integrins [64]. However, their expression remains unchanged upon UBC9 silencing (Figure 3B). Thus, inhibition of the SUMO pathway in addition to potentiate NK cell-mediated recognition and killing of CD155 positive tumor cells [63] also promotes the CD155-mediated adhesion of MM cells to stromal cells (Figure 3C). 

Even though CD155 expression is recognized as a danger signal by cytotoxic lymphocytes, reduced DNAM-1 levels were found on the surface of NK cells from peritoneal fluids of ovarian carcinoma patients as a consequence of chronic stimulation by CD155-bearing tumor cells [50]. Moreover, in Acute Myeloid Leukemia patients, CD155 and Nectin2-expressing leukemic blasts induces DNAM-1 down-modulation leading to an impairment of NK cell cytotoxicity [65]. In line with these results, NK cells derived from patients with advanced MM show low DNAM-1 expression respect to precancerous stages [51,66]. Accordingly, DNAM-1 has been shown to be up-regulated in T lymphocytes derived by CD155 deficient mice [67]. Relevant to this, CD155 expression on tumor-infiltrating myeloid suppressive cells induces DNAM-1 down-modulation from the surface of NK and T lymphocytes and impairs their ability to reject CD155-positive transplanted tumors [34]. 

All these findings provide evidence that prolonged exposure to CD155 promotes DNAM-1 down-modulation leading to an impairment of NK and T cell cytotoxicity. 

### 3.2. Inhibitory CD155 Receptors: TIGIT and CD96

Accumulating data demonstrate that in advanced tumor stages two inhibitory receptors structurally related to DNAM-1 are up-regulated on NK and cytotoxic T cells: T-cell immunoreceptor with immunoglobulin and ITIM domains (TIGIT) and T cell-activated increased late expression (Tactile), also known as CD96 [68]. They both share with DNAM-1 the ability to bind CD155 but contain an ITIM motif that can transduce inhibitory signals and counterbalance the DNAM-1 mediated activating signals [46,69]. Moreover, they show a higher affinity for CD155 than DNAM-1 [70,71]. 

Regarding mouse CD96 (mCD96), it appears that it mainly controls the extent of cytokine production by NK cells that critically depends on an interaction with the mature dendritic cells (DCs) [46] while direct killing tested in vitro is almost unaffected. Moreover, Smyth’s group demonstrated that disrupting the interaction between mCD96 and mCD155 by using anti-CD96 blocking antibodies, metastatic spread was inhibited in several tumor models [72]. Interestingly, despite this evidence documenting the involvement of mCD96 as an inhibitory NK cell receptor, human CD96 (hCD96) was initially described as an NK cell activating receptor [73]. However, NK cell efficiency to kill in vitro CD155 positive ovarian carcinoma cells [50] or myeloma cell lines [51] was not affected in the presence of neutralizing anti-CD96 monoclonal antibodies. Thus, whether hCD96 activates human NK cells is still a matter of debate. 

The functional differences between human and mouse CD96 may reflect their structural diversity: Only the cytoplasmic tail of human CD96 contains a YXXM domain that has the capability to recruit the p85 subunit of PI3 kinase [74]. Moreover, in human but not in mouse, two splice isoforms of CD96 exist that differ in their Ig-like extracellular domains and their binding affinity to hCD155 [75]. 

Unlike CD96, the role of TIGIT as an inhibitory NK cell receptor is well established in both humans and mice [69,71,76] and several mechanisms may explain TIGIT mediated inhibition [71,76,77,78,79]. First, TIGIT is able to disrupt DNAM-1 binding to its ligands on both T and NK cells [71,77]. Furthermore, it has been proposed that TIGIT can also directly bind to DNAM-1 *in cis* interfering with its homodimerization and blocking its co-stimulatory function in T cells [78]. Finally, upon interaction with CD155, the ITIM domain of TIGIT directly recruits SHIP-1 that counteracts activating signals, thus impairing NK cell-mediated cytotoxicity and IFN-γ production [76,79]. While the ITIM domain is exclusively responsible for TIGIT inhibitory function in human [69], additional signals are induced by murine TIGIT in NK cells and involve the ITT domain in the cytoplasmic tail, which indirectly activates SHIP-1 and inhibits PI3K-mediated signaling [76,79]. 

Both TIGIT and CD96 are significantly up-regulated on chronically stimulated tumor-infiltrating NK and T cells, representing markers of exhausted cytotoxic cells [78,80,81]. Indeed, their blockade achieved with monoclonal antibodies prevents exhaustion and promotes NK cell effector functions in murine models of tumor progression [80,82]. Of note, high expression of hCD96 on cells within the tumor microenvironment correlates with poor prognosis and resistance to chemotherapy [83,84], indicating hCD96 as a diagnostic marker. Finally, the expression of TIGIT correlates with functional NK and T cell impairment and poor prognosis in several types of cancers [85,86,87]. 

All together, this evidence demonstrates that during tumor progression the balance between DNAM-1 and its inhibitory counterparts is deregulated by an up-regulation of TIGIT and CD96, and a concomitant decrease of DNAM-1 expression. Therefore, in advanced clinical stages CD155 may contribute to dampen NK and T cell activation by the engagement of inhibitory receptors, favoring immune escape. Concurrently, CD155 could trigger intracellular signals in tumor cells upon interaction with its receptors, as formally demonstrated in DCs in which TIGIT-mediated CD155 engagement induces IL-10 secretion [70]. 

Regardless, both CD155 and its receptors represent promising targets for cancer immune therapy aimed to prevent exhaustion of tumor infiltrating lymphocytes [82]. In this context, six anti-TIGIT antibodies have entered clinical trials due to the promising results obtained in preclinical studies [85].

On the other hand, different chemotherapeutic drugs have been shown to increase CD155 expression and NK cell responsiveness, thus representing potential strategies aimed to improve tumor immune surveillance [88,89].

## 4. Concluding Remarks

CD155 is an adhesion molecule up-regulated during tumor progression that may favor tumor cell proliferative and migrating ability.

On the other hand, CD155 represents a danger signal for the activating receptor DNAM-1 expressed on NK and T cells being implicated in tumor cell recognition and killing. Examples of tumor strategies aimed to evade DNAM-1 recognition support the idea that in early phases of malignant transformation the up-regulation of membrane CD155 isoforms could be a potential tool to improve the ability of immune cells to fight cancers. To this regard, it can be useful to clarify whether DNAM-1 could equally recognize the different transmembrane isoforms with the aim to preferentially up-regulate the CD155δ isoform that is unable to trigger pro-tumorigenic signals. However, it is important also to consider that tumor progression facilitates the expression of the inhibitory receptors TIGIT and CD96 that compete with DNAM-1 for CD155 binding and dampen cytotoxic response. Therefore, in advanced clinical stages, strategies aimed to improve CD155 expression need to be associated with the use of blocking antibodies for inhibitory receptors in order to selectively promote DNAM-1 activating signaling in cytotoxic lymphocytes.

A better understanding of CD155 role as well as of the molecular mechanisms underlying CD155 up-regulation on transformed cells may lead to the development of new therapeutic strategies aimed to improve immune response against tumor cells.
